# Drivers׳ merging behavior data in highway work zones

**DOI:** 10.1016/j.dib.2016.01.047

**Published:** 2016-01-30

**Authors:** Mahmoud Shakouri, Laura H. Ikuma, Fereydoun Aghazadeh, Sherif Ishak

**Affiliations:** a School of Civil and Construction Engineering, Oregon State University, Corvallis, OR 97331, USA; b Department of Mechanical & Industrial Engineering Louisiana State University, Baton Rouge, LA 70803, USA; c Department of Civil and Environmental Engineering, Louisiana State University, Baton Rouge, LA 70803, USA

**Keywords:** Work zone, Merging behavior, Subjective workload, Safety

## Abstract

There have been growing research interests in finding a suitable work zone layout to improve work zone safety and traffic efficiency. This paper contains data supporting the research article entitled: Effects of work zone configurations and traffic density on performance variables and subjective workload (Shakouri et al., 2014 [1]). A full factorial experiment was conducted to compare the efficiency of two work zone configurations by using a driving simulator with two levels of work zone configuration, two levels of traffic density and three levels of sign placement as fixed factors. Seven female and 23 male participants completed the experiment. In this paper we present the data relating to demographic information of participants, driving simulator data and subjective workload evaluation of participants for each work zone.

**Specifications Table**

**Table:** 

Subject area	*Civil Engineering*
More specific subject area	*Transportation*
Type of data	*Tables*
How data was acquired	*Simulation and questionnaire*
Data format	*Modified*
Experimental factors	*Merge configuration, traffic density, and sign placement*
Experimental features	*Participants drove in a driving simulator. Driving performance was collected automatically by the simulator. After each drive, participants filled a NASA-TLX questionnaire.*
Data source location	*Baton Rouge, LA, US*
Data accessibility	*Data is provided in Supplementary materials directly with this article*

## Value of the data

• Drivers׳ behavior and performance data in work zones are essential to identifying the strengths and weaknesses of highway work zones.
• NASA-TLX data provides feedback on drivers׳ experienced workload in highway work zones.
• Physical, cognitive, and task analysis data can be used to make models to explain drivers׳ behavior in high work zones and improve work zone safety and performance.

## 1 Data

• Table 1 shows the full factorial design used in this study. Each participant drove 12 scenarios which were randomized by Latin Square technique. 30 People participated in this experiment and their demographic information is provided in “Demographic” sheet in Supplementary materials.
• Fig. 2, Fig. 3 show the layouts of work zones. Each work zone was divided into five zones. Driving behavior data for each zone is given in “Performance” sheet in the supplementary materials.
• After each 12 scenario, a NASA-TLX questionnaire was given to the participant to rate his/her subjective workload. These data are given in “TLX” sheet in the supplementary materials.

## 2 Experimental design, materials and methods

**Fig. 1:**
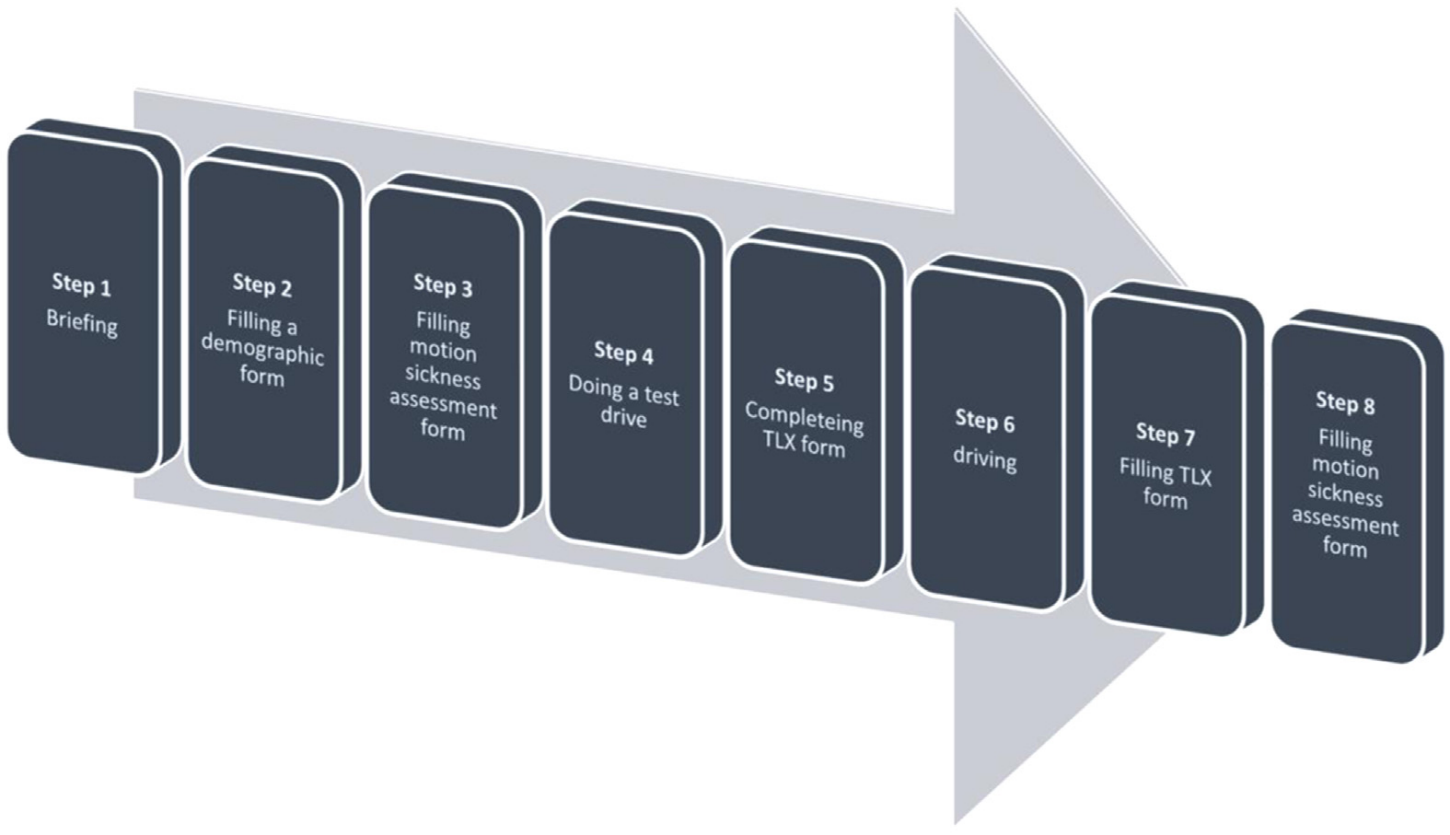
Summary of experiment procedure.

**Fig. 2:**
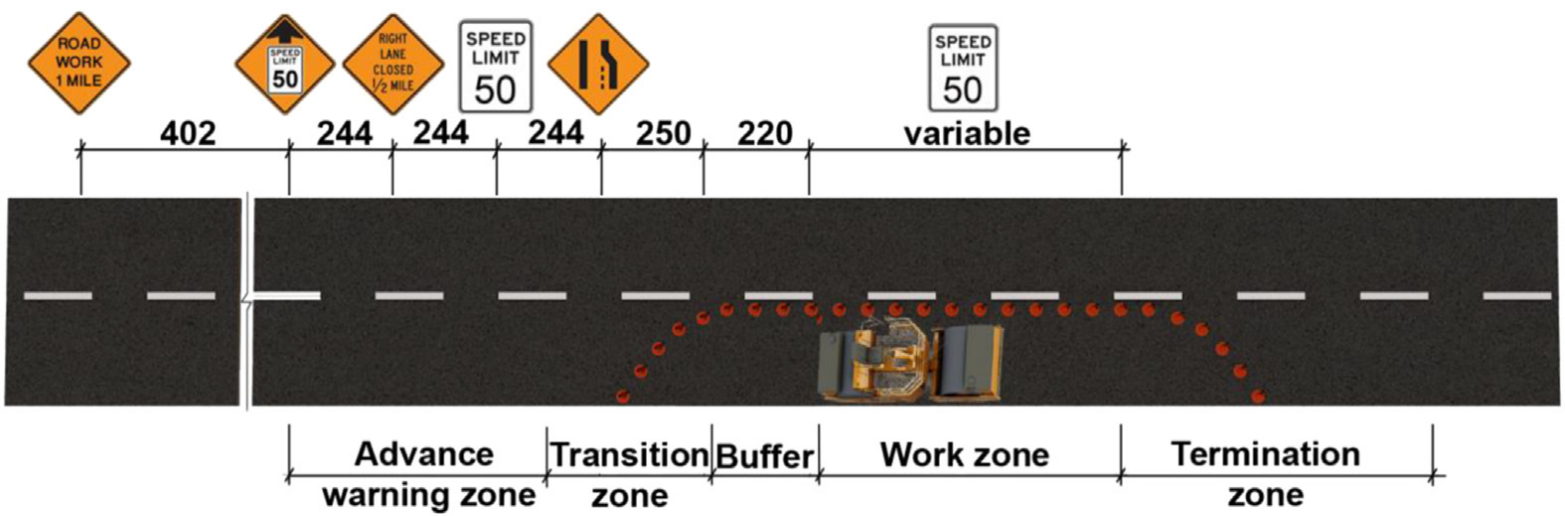
Conventional lane merge layout suggested by U.S. Department of Transportation [2] (standard dimensions in meter).

**Fig. 3:**
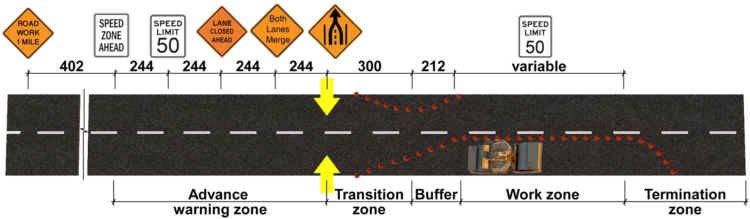
Joint lane merge layout suggested by Idewu and Wolshon [3] (standard dimensions in meter).

A 2×3×2 within-subjects factorial design with work zone, sign placement, and traffic density as independent variables was used in this study. Table 1 shows the experiment design and the corresponding levels of each fixed factor. An on road high-fidelity driving simulator [4] was used in this study to simulate driving through work zones. Fig. 2, Fig. 3 show the layout of the two work zones. Each work zone was divided into five zones and driving behavior data was collected for each zone. A demographic questionnaire containing 11 items was used to collect data regarding gender, years of driving experience, annual driving mileage and whether the participant was involved in an accident or received a traffic ticket for violating traffic rules. Subjective workload was measured by using NASA-TLX [5]. Participants in this study were recruited through convenience sampling from Louisiana State University. Seven female and 23 male students participated in the study. Fig. 1 shows the steps taken to conduct the experiment. After each scenario, participants filled a TLX form. Therefore, steps 6 and 7 repeated 12 times. Step 8 was repeated after each three drives to make sure that participants were feeling well during the experiment.

## Figures and Tables

**Table 1:** Full factorial experiment design.

**Work zone**	**Sign placement**	**Traffic density**
CLM	25% Reduction	Low
High
Standard	Low
High
25% Increase	Low
High
JLM	25% Reduction	Low
High
Standard	Low
High
25% Increase	Low
High

## Footnotes

Appendix A Supplementary data associated with this article can be found in the online version at doi:10.1016/j.dib.2016.01.047.
