# Supplementary material for: Drivers׳ merging behavior data in highway work zones
Source: Data Brief. 2016 Jan 30;6:829–32. doi: 10.1016/j.dib.2016.01.047 (PMC4749935; doi:10.1016/j.dib.2016.01.047)
Supplement: Supplementary file 2 — Supplementary material [file mmc2.pdf]

Demographic Information Form

Participant id -------------------

Date of Experiment -------------   Time of Experiment -----------------

Instruction: Please fill an appropriate box for each question.

1. Gender ☐ Male ☐ Female

2. Age ☐ <20 ☐ 20-29 ☐ 30-39 ☐ 40-49 ☐ ≥50

3. How long have you had your driving license? -----------------

4. What is your driving experience?

   ☐ <1 ☐ 1-5 ☐ 5-9 ☐ ≥10

5. Estimate the number of miles you drive each year ---------------------

6. During the past year (12 months) have you been involved in any accidents?

   ☐ Yes ☐ No

7. If yes, how many accidents --------------------

8. During the past year (12 months) have you had any highway violations?

   ☐ Yes ☐ No

9. If yes, how many violations ----------------------------

10. How often do you talk on your cell phone when you drive?

    ☐ Never ☐ Sometimes ☐ Always

11. How often do you text message when you drive?

    ☐ Never ☐ Sometimes ☐ Always
