# Supplementary material for: Drivers׳ merging behavior data in highway work zones
Source: Data Brief. 2016 Jan 30;6:829–32. doi: 10.1016/j.dib.2016.01.047 (PMC4749935; doi:10.1016/j.dib.2016.01.047)
Supplement: Supplementary file 5 — Supplementary material [file mmc5.pdf]

Participant id ------------------- Merge Type: --------------------------

Date of Experiment ------------- Time of Experiment ------------------

NASA-TLX Descriptions

Refer to these descriptions as you complete the Workload Rating sheet.

Mental Demand: *Low/High* How much mental and perceptual activity was required (e.g. thinking, deciding, calculating, remembering, looking, searching, etc.)? Was the task easy or demanding, simple or complex, exacting or forgiving?

Physical Demand: *Low/High* How much physical activity was required (e.g., pushing, pulling, turning, controlling, activating, etc.)? Was the task easy or demanding, slow or brisk, slack or strenuous, restful or laborious?

Temporal Demand: *Low/High* How much time pressure did you feel due to the rate or pace at which the tasks or task elements occurred? Was the pace slow and leisurely or rapid and frantic?

Performance: *Excellent/Poor* How successful do you think you were in accomplishing the goals of the task set by the experimenter (or yourself)? How satisfied were you with your performance in accomplishing these goals?

Effort: *Low/High* How hard did you have to work (mentally and physically) to accomplish your level of performance?

Frustration Level: *Low/High* How insecure, discouraged, irritated, stressed, and annoyed versus secure, gratified, content, relaxed, and complacent did you feel during the task?

Instructions: select the member of each pair that provided the most significant source of workload variation in these tasks. (This page is given to participants after the test drive)

#	Physical Demand	Mental Demand
1	Temporal Demand	Mental Demand
2	Temporal Demand	Physical Demand
3	Performance	Physical Demand
4	Temporal Demand	Frustration
5	Temporal Demand	Effort
6	Performance	Mental Demand
7	Frustration	Mental Demand
8	Effort	Mental Demand
9	Frustration	Physical Demand
10	Effort	Physical Demand
11	Temporal Demand	Performance
12	Performance	Frustration
13	Performance	Effort
14	Effort	Frustration

Workload Rating

Instructions: Place a vertical mark on each scale that represents the magnitude of each factor in the task you just performed. (This page is given to participants after each drive)

Mental Demand

Low High

Physical Demand

Low High

Temporal Demand

Low High

Performance

Excellent Poor

Effort

Low High

Frustration Level

Low High
